# Through thick and thin: Russia, China and the future of Eurasian International Society

**DOI:** 10.1057/s41311-020-00260-6

**Published:** 2020-07-14

**Authors:** Zachary Paikin

**Affiliations:** School of Politics and International Relations, University of Kent, Canterbury, CT2 7NX UK

**Keywords:** Russia, China, Eurasia, Central Asia, English School, International order

## Abstract

Recent years have seen Moscow and Beijing driven closer together as their respective relations with Washington have deteriorated. While a normative convergence between Russia and China appears to be underway, there remains a legacy of historical mistrust in addition to potential irritants that could plague their bilateral relationship. Nonetheless, if Western sanctions against Russia and pressure on China continue unabated, they may have little option but to continue their rapprochement. Against this backdrop, this article will explore whether the two countries currently display a ‘thin’ or ‘thick’ bilateral relationship, making use of an English School theoretical framework. It will do so by exploring established English School concepts such as pluralism and solidarism and discussing notable literature within the School on contestation in order to derive conclusions regarding the potential future shape of international order in Eurasia.

## Introduction

Recent years have seen Moscow and Beijing driven closer together as their respective relations with Washington have deteriorated (Cox 2017). Many experts now characterize their strategic partnership as an ‘entente’ (Trenin 2018; Kortunov 2019), featuring ‘a common understanding of global processes and a similar vision of the future of world order’, pointing to a significant normative convergence between the two powers (Lukin 2018: 63). While there remains a legacy of historical mistrust in addition to various irritants that could plague their bilateral relationship, if Western sanctions against Russia and pressure on China continue unabated then they may have little option but to continue their rapprochement (Bolt and Cross 2018: 96). Deepening Sino-Russian ties contrast with a global order that has been described as ‘thin’, with rival great power interests and norms becoming increasingly apparent as the post-Cold War era progressed (Ikenberry 2018). Bull and Watson (1984: 430) note that states in contemporary global international society are ‘less united by a sense of common interest in a framework of rules and institutions governing their relations with one another’ than the European powers of past centuries, despite ‘an immense growth of international law, diplomatic representation, and international organization’ since the end of World War II.

In the years following the Russian annexation of Crimea, having failed to realize its initial post-Cold War vision of a ‘Greater Europe’ from Lisbon to Vladivostok, Moscow announced plans for a ‘Greater Eurasia’ instead—a supercontinent-wide integration project with Russia at its centre (Karaganov 2016; Lo 2019a). Europe is free to join this Greater Eurasian configuration so long as it agrees to uphold its pluralistic principles (Lukin 2018: 187). This raises important questions surrounding how to define a ‘thin’ order or relationship versus a ‘thick’ one. Are there circumstances under which one could conceive of a pluralistic international order as ‘thick’? This article seeks to explore whether the contemporary Sino-Russian relationship can be considered as ‘thick’ from an English School perspective. Although not constitutive of ‘Greater Eurasia’ as a whole, a conceptual exploration of their bilateral ties and strategic aims can illuminate important elements of the emerging international order among Eurasian actors. It will begin by exploring the traditional English School concepts of pluralism and solidarism in addition to other contrasts within the School to provide a picture of how the School would conceive of thin and thick social relationships. It will then probe the contours of Russia–China ties to derive conceptual conclusions regarding the nature of ordering practices in contemporary international society.

## ‘Thick’ and ‘thin’: why the English School?

Launched in the years following the 2013–2014 Ukraine crisis, Russia’s ‘Greater Eurasia’ vision remains unformed in many ways, possibly representing an attempt to stall for time and position Moscow as an equal architect of the emerging Eurasian order (Lo 2019a; Paikin et al. 2019). Nonetheless, it is noteworthy that three of the five land corridors proposed by China’s Belt and Road Initiative (BRI) traverse Russia and/or post-Soviet Central Asia: the China–Mongolia–Russia Economic Corridor, the New Eurasia Land Bridge Economic Corridor and the China–Central Asia–West Asia Economic Corridor (Ramasamy et al. 2017). Therefore, while the respective visions proposed by Moscow and Beijing may span the Eurasian supercontinent (and beyond), it is possible to conceive of the Sino-Russian partnership as lying at the core of the fledgling Eurasian order, at the intersection of the BRI and the Russian-backed Eurasian Economic Union (EAEU) (Valdai Discussion Club 2015; Bordachev 2018: 70).

The English School of international relations is a useful tool for studying periods of change in the international order, in part due to its focus on questions surrounding the legitimacy of given norms (Stivachtis 2017; Clark 2011: v) but also due to the ontology of states possessed by its signature concept of ‘international society’ (Wight 1977: 12; Buzan 2014: 89). Buzan (2012: 36) defines an international society as ‘the institutionalization of shared interests and identity among states and the creation and maintenance of shared norms, rules, and institutions among them’. Similarly, Wight (1977: 13) characterizes it as a common set of norms that ‘condition [states’] behaviour and identity’. The relationship between the notions of international order and international society is also discussed at length in English School canon. Flockhart (2016) appears to use the terms almost interchangeably at times, seeing as both are present at any given moment in the global or regional political systems.^1^ Sakwa (2017: 42–47) draws a sharper distinction between order and society by claiming that they constitute two separate ‘levels’ of an overall global political system, but that nonetheless interact with each other in what he calls the ‘sphere of norms’. Reus-Smit and Dunne (2017: 31–32), disagreeing with Bull’s characterization of an international order as a ‘pattern of activity that sustains the elementary or primary goals’ of international society, contend that a breakdown of order is not merely a failure to realize those goals but rather of ‘the rules and institutions that constitute [international] society’. All these definitions point to the existence of a close relationship between the concepts of international order and international society, which has been acknowledged explicitly in recent English School scholarship (Reus-Smit and Dunne 2017: 31–32). This is reflected as well in definitions of the concept of ‘order’ itself, which—like definitions of international society—often incorporate the notion of norms. For instance, Lebow (2018: 305–306) denotes order as ‘legible, predictable behaviour in accord with recognized norms’.

Recent English School scholarship contends that mere mutual recognition is inherently social and that therefore there can be no distinction between ‘international system’ and ‘international society’ (Reus-Smit and Dunne 2017: 31).^2^ This suggests that one need not concern oneself with whether Sino-Russian relations have crossed over from the purely regulatory into the social realm. Therefore, there exists a basis to explore the Sino-Russian relationship, in their shared Central Eurasian neighbourhood as well as in the wider Eurasian space, from a social perspective. Developing further the English School understanding of ‘thick’ and ‘thin’ stands to provide an alternative analytical view on the shape of the changing global order and its various sub-orders, which may vary along geographical but also identity-based lines (Flockhart 2016). Such an undertaking is worthwhile given recent conceptualizations of ‘thick’ and ‘thin’ by more mainstream scholars. For instance, Mearsheimer, a neo-realist scholar, recently contends:

A thick or robust order comprises institutions that have a substantial effect on state behaviour in both the economic and military realms. […] A thin order, on the other hand, can take three basic forms. First, it might deal with only the economic or military domain, but not both. Even if that realm contained strong institutions, it would still be categorized as a thin order. Second, an order might deal with one or even both realms, but contain weak institutions. Third, it is possible, but unlikely, that an order will be involved with economic and military matters, but will have strong institutions in only one of those areas (Mearsheimer 2019).

Whether a stark delineation between security and economic affairs should be employed as the means of separating ‘thick’ from ‘thin’ is a matter of contention and will be returned to below. What is more, Mearsheimer’s analysis reveals an exclusive focus on secondary institutions instead of primary ones. Debate over the legitimacy of certain interpretations of primary institutions—including sovereignty, the balance of power and great power management—in global international society has become an even more central feature of international politics in recent years (Allison 2013; Lind and Press 2020). Secondary institutions such as ‘shared practices and international organizations and regimes’ are fashioned to produce order by realizing the aims set out by specific interpretations of primary institutions, which are cardinal principles ‘essential for producing and maintaining order’ (Flockhart 2016). This is in line with the English School notion that ‘constitutional normative principles’ and ‘rules of coexistence’ are of greater significance to the foundations of any international society than ‘rules to regulate cooperation’ (Buzan 2014: 98). The difference between the English School and other approaches regarding how institutions should be understood highlights the importance of probing how the School would categorize ‘thin’ and ‘thick’ social relationships, given the role played by institutions in regulating conduct between states.

However, the Sino-Russian relationship at the heart of the Eurasian order should be distinguished from a clearly delineated regional international society. Buzan (2012: 22–25) defines a region as ‘a geographically clustered subsystem of states that is sufficiently distinctive in terms of its structure and process to be meaningfully differentiated from a wider international system or society of which it is part’, with the EU, for example, constituting ‘a distinctive and very highly developed form of regional international society’. With Russia’s population core lying in Europe and China’s being situated in East Asia, it is difficult to contend that an international order featuring these two powers is ‘geographically clustered’. In fact, the secondary nature of Russian and Chinese interests in Central Eurasia may be a driving factor of both countries’ efforts to de-securitize their relationship, in order to strengthen their ability to pursue their core interests in Eastern Europe and East Asia, respectively (Kofman 2019). Nor is the purported scope of the Greater Eurasia and BRI visions geographically clustered. Moreover, the Central Asian states that lie at the intersection of the EAEU and BRI have a preference for pursuing multi-vectored foreign policies with a plethora of external actors (Cooley 2012: 68).

What is more, unlike the member states of the EU, Russia and China are great powers at the global level capable of checking the military power of the USA, having formed part of Kissinger’s famous ‘triangular diplomacy’ during the Cold War (Kofman and Connolly 2019). Buzan and Wæver (2003: 435–436) contend that Russia’s regional ambitions in the post-Soviet space serve an instrumental purpose, namely, to strengthen Moscow’s claim to great power status at the global level. Kaczmarski (2017) notes that efforts to harmonize the EAEU and BRI amount to ‘promoting regionalism with [Russia and China’s] direct participation’, differing from more traditional regional bodies such as the EU and ASEAN which have flourished under a great power’s umbrella. The Sino-Russian strategic partnership spans contexts of varying strategic and geographical scope, ranging from efforts to promote security and development in their shared neighbourhood in Central Asia, to their differing but also overlapping approaches to engaging with leading Eurasian players such as India, Japan and ASEAN, to their combined balancing against the USA.

## ‘Thick’ and ‘thin’ in contemporary international society

Recent English School scholarship has expounded on how great powers project their preferred norms onto international society in an effort to shape or defend its structure (Sakwa 2017: 44–47; Paikin 2020). Therefore, rather than discussing the ‘thickness’ of any clearly delineated regional society, the pertinent question concerns the thickness of the Sino-Russian relationship as an ‘anchor’ for the wider Eurasian order and society. Yet unlike the Western community of liberal states, Russia and China’s political systems—while sharing certain authoritarian features—differ significantly. If uniformity of political systems and a strong degree of shared culture and values (rather than merely shared norms) is a requirement of a ‘thick’ society, then the Sino-Russian compact does not meet it. To use more established English School vocabulary, is normative agreement between Russia and China sufficient to provide their ‘pluralist’ relationship with a ‘thick’ character, or rather must it move in a more ‘solidarist’ direction for this to be the case? This, in turn, depends on how these two core English School concepts—pluralism and solidarism—are interpreted. While solidarism is usually taken to emphasize elements of convergence and homogeneity in international society, the reality may be more complex (Linklater and Suganami 2006: 146).

Although recent English School scholars have argued against a distinction between systemic and social politics in practice, drawing a conceptual line between the notions of ‘system’ and ‘society’ can serve as a useful analytical tool. While actors in an international system merely recognize one another’s empirical existence and are ‘fortuitous’ when a balance of power is reached, when the level of a pluralist international society is attained they develop norms such as respect for one another’s sovereignty and establish an equilibrium that is ‘contrived’, thus rendering pluralism ‘a qualified progressivist interpretation of world politics […] in which states move beyond purely strategic forms of interaction’ (Linklater and Suganami 2006: 130–135). In other words, there is a shared respect for the pluralistic character of the society. The absence of a trend towards homogeneity does not imply that it is devoid of normative content. Linklater and Suganami (2006: 153) have therefore concluded that states are capable of working out a form of ‘universal solidarity’ to legitimate the global economic and political order that is rooted in ‘a set of values and standards which do not require conformity with some vision of the good life which may be preferred in the most powerful society’ and ‘which reveal that very different societies can agree on forms of human solidarity in the context of radical cultural and religious differences’.

Moreover, the states spread by European powers across the globe took the form of those developed in the nineteenth century rather than the early modern period, by which point European international society’s pluralist foundations had already been placed under stress by the advent of nationalism, thus generating a link between pluralism and solidarism (Buzan 2014: 97–112).^3^ This is also in line with Sakwa’s (2017: 49) assertion that any global order features elements of both pluralism and solidarism. The institution of sovereignty, traditionally linked with the preservation of international society’s pluralist character due to its association with Westphalia and the norm of non-intervention, has acquired certain solidarist characteristics as it has been extended to all peoples on the principle of sovereign and human equality (Buzan 2014: 141). Therefore, the institutions and norms of contemporary sub-orders within the global order cannot be considered exclusively pluralist in their character,^4^ and even if they could, this would not necessarily imply that the order in question is ‘thin’. In short, pluralist societies are often ‘thicker’ than originally imagined and solidarist ones do not necessarily trend towards political and ideological convergence. This contrasts with Buzan’s (2012: 38-45) fourfold characterization that implicitly equates the thickness of international societies with increased solidarism—‘Power-Political’ (societies featuring states driven by survival), ‘Coexistence’ (rooted in a balance of power, sovereignty and diplomacy), ‘Cooperative’ (featuring a solidarist element but varying according to what values are shared, why and how) and ‘Convergence’ (with states possessing similar forms of government)—and even notes that the strength of the society depends on its ‘degree of cultural unity’ and the ‘types and mixtures of states’ to be found within it. Such a framework is at odds with the fact that the advance of solidarism and the proliferation of formalized institutions over the course of the post-war period have not produced a thick global international society (Bozeman 1984: 404; Lebow 2008: 498).

Exhibiting somewhat of a parallel with more recent English School scholars, Watson (1992: 318) contends that a ‘society that goes beyond rules and institutions to shared values and assumptions’ has ‘hitherto always developed within a cultural framework’. A view with similar implications is also advanced by scholars outside the English School, including Mearsheimer (2019) who predicts that thick bounded orders will consolidate around the USA and China, while allowing for the continued existence of a thin global order ‘concerned mainly with overseeing arms control agreements and making the global economy work efficiently’. However, unlike Watson, this latter view is blind to the question of culture and therefore sees fewer impediments to the establishment of a deep Sino-Russian relationship. The view that geographically bounded orders will be largely constitutive of global international society is in line with Flockhart’s notion (2016) of a ‘multi-order world’, although her contention allows for a plurality of orders rather than just two. This differs somewhat from Sørenson’s claim (2016: 20) that international society is not headed for a ‘world of regions’ but merely ‘a more decentred world’, which hints that the division between Russia and China need not necessarily be stark.

English School scholars also disagree on the nature and effect of contestation. Bozeman (1984: 405) contends that key norms and institutions such as state sovereignty have become ‘critically impaired’ as European international society expanded into a culturally diverse global context. Watson (2007: 78) notes that the gap between the practice and theory of international society—including the reality of an integrated world and the continued emphasis on the Westphalian state as the legitimate unit actor—has produced a contradiction significant enough to require an adjustment of its legitimately agreed-upon norms and rules. This differs from more recent accounts that cast international society’s normative evolution in a less critical light (Reus-Smit and Dunne 2017: 29). Rather than highlight international society’s normative dilution as it expanded from Europe to encompass the globe, Phillips (2017: 60) contends that the contemporary global international society was formed through a ‘polycentric pattern of expansion’, with Europe only eclipsing the rest at the time of the Industrial Revolution rather than the Age of Exploration. Buzan (2017: 246) considers the evolution of the norm of sovereignty through the centuries to be a form of ‘dynamism’ rather than dilution. Rather than evidence of any fundamental tension, certain regions could simply lack some of global international society’s primary institutions or perhaps interpret them differently to generate ‘significantly different practices associated with them’ (Buzan 2012: 37).

Despite instances of agreement, the above analysis confirms that competing views exist within the English School regarding the extent to which normative divergence impedes the potential for thickness. While Little (1999: 22–26) contends that rival ‘universalizing ideologies’ contributed to the erosion of international society’s ‘societal balance of power’ over the past two centuries, Reus-Smit and Dunne (2017: 36) view contestation as ‘an engine of international social development’. However, the more recent English School accounts highlighted above, while not necessarily casting normative rivalry in a negative light, privilege the process of evolution and change in their analysis, often occurring over decades and even centuries. This differs from an analysis aimed at evaluating where an international order or society is situated at a given moment in time, even though it remains useful for speculating about the consequences of normative rivalry. Additionally, Watson’s aforementioned remark concerning societies of shared values and monoculturalism may not apply to the case of the Sino-Russian relationship, as shared norms differ from shared values. One can therefore conclude that an interstate relationship rooted in shared norms in the context of a purely pluralist international society can be considered ‘thick’, to say nothing of a society that features elements of both pluralism and solidarism, but that this thickness could be impaired by contestation over certain norms and primary institutions according to certain perspectives within the School.

## Russia–China relations today

Following the onset of the Ukraine crisis in 2014, Moscow accelerated its previously declared ‘pivot to the east’ (Bolt and Cross 2018: 12). In part due to the descent of its relations with the West into outright rivalry, Russia’s engagement with Asia has risen to its highest and most serious level since the end of the Cold War. Ukraine’s decision to sign an Association Agreement and establish a Deep and Comprehensive Free Trade Area with the EU prodded the EAEU into a more Central Asian focus, while Moscow also began to outline its vision of an integrated, supercontinent-wide ‘Greater Eurasian Partnership’. Over the past several years, Russia and China have undergone what appears to be a genuine normative rapprochement, having been brought together by shared scepticism of Western interventionism and democracy promotion (Paikin et al. 2019). Instances of perceived norm violation by Western states in Kosovo, Iraq and Libya followed by the more recent apparent American strategy of ‘dual containment’ vis-à-vis Russia and China have expedited the deepening of relations between Moscow and Beijing (Trenin 2019a). As one long-term observer of the bilateral relationship puts it, Russia and China now ‘agree on most international issues’ (Lo 2019b). Kaczmarski (2019a) elaborates on this, identifying ‘several points of convergence’ between the two powers, including ‘common views on the evolution of international order; emphasis on the centrality of the UN; common attitudes towards global and regional security challenges; and joint approaches towards multilateral cooperation in international affairs’. Moreover, the military and security dimension of the relationship has seen a substantial increase in bilateral and multilateral cooperation—ranging from within the SCO to the Vostok-2018 and Tsentr-2019 military exercises—a fact made even more remarkable considering the power transition between the two that has occurred over the past several decades. Although the official line is that both sides are pursuing a strategic partnership (Lukin 2018: 62), the term ‘alliance’ has been used to describe their relationship, albeit an informal one (Kashin 2019; Lukin 2020).

Moscow and Beijing’s nominally joint commitment to the principle of non-interference in the internal affairs of sovereign states is not always evident in practice, with Russia notably having violated the territorial integrity of Georgia, Moldova and Ukraine while also meddling in Western elections. That said, both sides have implicitly recognized the notion of spheres of influence, with Beijing deferring to Russian interests in Eastern Europe and the Middle East and Moscow acknowledging Chinese pre-eminence in East Asia (Lukin 2020), further underlining their normative rapprochement. Nevertheless, while Moscow and Beijing agree on broad concepts and big-picture norms such as anti-hegemonism, this is not the same as forging a deeper alliance to establish a comprehensive set of post-Western norms and institutions (Lo 2019b). A more sceptical view would assert that both parties are brought together largely by their opposition to American hegemony and that a more definitive abandonment by Washington of its leadership role in the liberal international order could lead to the resurrection of a Yalta-type order (Lo 2019a). However, it remains doubtful whether Russia is able to turn its back on an increasingly powerful China considering the state of Russia–West relations (Gabuev 2020a), to say nothing of the difficulty of fully resurrecting a Yalta-type order in an era of increasingly diffuse global power (Horsman and Marshall 1995: 167).

Against this backdrop lies the perennial question of Russia’s great power status and the recurring theme of its desire to be treated as an ‘equal’ (Liik 2019). Russia sought to secure this status throughout the bulk of the post-Cold War period by joining a transformed Euro-Atlantic community and building a common European space from Lisbon to Vladivostok (Trenin 2019b). Instead, Russia’s return to great power status—one of the core goals of the country’s post-communist regime—occurred in the context of a renewed confrontation with the West (Trenin 2019c). In other words, following a quarter-century of uncertainty, Russia appears to have concluded that it can only achieve its core international aims outside the West rather than by joining it. Russia is now entering a period of its history where it must begin to emphasize—if not privilege—its eastward foreign policy vector. However, this will present challenges as the country, despite having been subject to various Turkic, Mongolic and other ‘eastern’ influences throughout its history, is largely culturally and demographically oriented towards the West (Lo and Hill 2013). Although it is one of the leading states in Europe, it is likely to be relegated to the second tier of powers in the Asia–Pacific region (Kortunov 2018). This in itself is likely to force Russia to rethink how to interpret its ‘great power status’, with Trenin (2019d) calling this ‘a pivot by Russia towards itself, in search of a balancing point in a quickly changing global environment’ rather than a pivot to the east.

The question of Russia’s great power status can be thought of as relating to the traditional primary institutions of great power management and the balance of power. Therefore, if Russia and China prove unable to agree on the nature and implications of Moscow’s status as a great power in Eurasian and global affairs, then this may preclude the long-term entrenchment of a thick relationship between them. Lo (2019b) contends that Russia’s ‘Greater Eurasia’ vision was designed in part to breathe ‘new life’ into the EAEU after its failure to incorporate Ukraine and position it at the centre of the supercontinent-wide Eurasian integration process. Unlike other segments of Eurasia and the Asia–Pacific region, Central Asia lies within Russia’s historic sphere of influence. Moscow’s inability to ensure pre-eminence in its own ‘backyard’ may preclude the possibility of attaining global great power status, even as such status remains ‘central to [Russia’s] foreign policy identity’ (Cooley 2012: 51). Buzan and Wæver (2003: 435–436) similarly predict ‘dramatic’ effects at the domestic level should Russia ‘be forced out of its international [great power] role’. This reflects Flockhart’s (2020) assertion that ontological security is a key feature of resilient political orders, underscoring the extent to which perceived recognition of Moscow’s great power status remains crucial for stable Russia–China ties. That said, Russia may accept the inevitability of China’s increasing economic footprint in Central Asia, as Gabuev (2018) notes that ‘[d]eeper Chinese penetration into the region actually reduces incentives for [Central Asian] countries to seek export routes to Europe that might bypass Russia and create additional pressure on Russian exporters in its core market’. Both Russia and China have also thus far demonstrated a willingness to pursue non-exclusive goals there and play the region’s local rules (Cooley 2012: 162). However, this may suggest that Moscow’s relationship with Beijing is instrumental in nature, designed above all else to provide additional avenues through which it can counterbalance Western hegemony. Russia further diverges from China in the region regarding the purpose of the SCO, with the former preferring that it retain its focus on security and the latter striving to put economic issues on the agenda (Lanteigne 2018: 120).

Cooley (2012: 70) contends that no clear division of labour exists between the two powers in Central Asia, whereas others such as Umarov (2020) allow for a neater separation between Russian security responsibilities and Chinese economic clout. This raises the question of whether Russia and Central Asian states will grow increasingly economically dependent on China, a process that may be accelerated by the coronavirus pandemic (Gabuev 2020b). While these concerns are noteworthy, Lukin (2020) asserts that ‘economic asymmetry is not equal to political subordination […] Russia has been Europe’s resource periphery for centuries while acting politically as a great power. Why not repeat the same pattern with China?’ Moscow may have few options but to continue its strategic partnership with Beijing in the context of an increasingly hostile relationship with the West, but this is not necessarily identical to ‘vassal’ or ‘junior partner’ status. In addition to the identity-related issues flowing from Russia’s centuries of participation in European international society, the diametric opposition between a USA that sees pre-eminence in Europe as essential to its national security and a Russia that has clear interest in shaping the European security system helped to foster fears in Moscow of relegation to ‘junior partner’ status after the Cold War (Sushentsov and Wohlforth 2020). These fears were exacerbated by Washington and Moscow’s position as the pre-eminent powers in the Euro-Atlantic region, whereas the distribution of power is more diffuse in the pan-Eurasian context, allowing Russia to retain a sense of great power status more easily.

While Russia has grown increasingly dependent on China, the reverse has also become true as USA–China relations have grown fraught in recent years (Lukin 2020). The need for amicable ties with Moscow owes itself partly to a desire to avoid encirclement by the USA and its allies and partners (Lukin 2018: 46). Rather than generating a relationship of zero-sum dependence, Beijing’s growing presence in Central Asia has allowed the region’s states to ‘address development and security challenges that would otherwise be under-resourced’, while China has eschewed efforts to fashion a regional order from the top down (Thornton 2020; Cooley 2012: 173–174). Moreover, far from creating a Beijing-centric order, Khanna (2019) contends that investment across Eurasia by way of the BRI is likely to fuel growth across the supercontinent, thus leading to its gradual multi-polarization. That said, many Chinese policy experts view the SCO as functioning ineffectively, thinking of it as a mere ‘supplementary tool’ and thus casting doubt on Beijing’s commitment to multilateralism in key Eurasian theatres (Lukin 2018: 58). This reflects China’s general preference for conducting interstate relations bilaterally, with notable exceptions (Shambaugh 2013: 24). Nonetheless, Sino-Russian cooperation has allowed both Moscow and Beijing to appear to benefit from peaceful development, win–win diplomacy and the de-unipolarization (or ‘democratization’) of international relations, all of which are ‘key tenets of Chinese foreign policy’ (Wishnick 2010: 56). Even in the absence of ‘thick’ and robust secondary institutions, this bodes well for the future of the Russia–China strategic partnership from a normative perspective.

When it comes to the wider Eurasian region, Moscow and Beijing have notable foreign policy differences, including, for example, Russia’s desire to diversify its partnerships and maintain good relations with Vietnam despite the latter’s rivalry with China in the context of the South China Sea (Bolt and Cross 2018: 2). However, when it comes to relations with some of Eurasia’s larger powers, the Russia–China relationship is buttressed by a self-reinforcing logic. For example, New Delhi’s ties with Beijing have been marked by instances of both competition and cooperation, with India remaining the largest borrower from the Chinese-backed Asian Infrastructure Investment Bank despite the securitized nature of their bilateral relationship (Iwanek 2019; Rajagopalan 2020). If this ‘balancing act’ continues, then it could lead to the entrenchment of ‘thick’ pluralism in the Eurasian space along the lines envisioned by Moscow’s Greater Eurasia project, strengthening Russian ontological security and therefore the normative and identity-related foundations of the Sino-Russian partnership.^5^ If recent border clashes between Chinese and Indian forces generate increasing security competition between Beijing and New Delhi, however, this will only increase China’s dependence on Russia. In other words, self-sustaining processes have been initiated that encourage the further cultivation of the Sino-Russian strategic partnership over the coming years. Even if certain irritants persist in the Russia–China relationship in Central Asia or even on the wider Eurasian scale, ‘Moscow and Beijing need each other on much bigger issues’ (Lukin 2020). Shared perceptions that identify Washington as the primary external security threat continue to drive Moscow and Beijing closer together, even on technological issues where Russia has historically been more sovereignty-conscious (Gabuev 2020a).

Yet despite the genuine normative convergence that has developed between Russia and China, it is largely centred around broad principles rather than specifics. Kortunov elaborates on this issue, writing:

Beyond bilateral ties, Russia–China cooperation often boils down to reactions to emerging crises, such as those in Syria, on the Korean Peninsula or in Venezuela. The two countries do their best to counter the attempts of the United States to undermine the sovereignty of independent countries, apply double standards to global politics, and use sanctions and trade wars. […] Russia and China often use the same terms to describe the future they desire: multipolarity, a post-Western world, the indispensability of the rule of law, the indivisibility of security, and so on. Sadly, however, most of these terms remain predominantly proclamatory; there is no concrete meaning to them. If you dissect any of these notions with the sharp scalpel of a depoliticized analysis, you will find numerous latent contradictions, internal conflicts, and incongruities (Kortunov 2019).

While deeper engagement across the Asia–Pacific region could be a sign of a more developed and refined ‘pivot to the east’, a convergence with China remains in line with the traditional Russian foreign policy aim of counterbalancing American hegemony and the Western-led liberal international order (Lo 2019b). This is bolstered by the fact that Moscow has sought to enmesh Beijing in multilateral bodies and processes in Eurasia (Pieper 2018), suggesting a desire to hedge against any potential hegemon and not just the USA, although Korolev (2016) notes that Russia’s posture of hedging towards China differs in nature from its more strenuous efforts to balance against the USA. Moreover, the EAEU and China have agreed on a trade deal, but it is non-preferential and can therefore be interpreted as an attempt to tie China into multilateral processes rather than a genuine effort by Russia to pursue pan-Eurasian integration. Nonetheless, the nominal desire expressed by both countries to coordinate their respective integration projects—the EAEU and the BRI—despite their differences is a significant one. With one of the major corridors of the latter passing through Central Asia, the potential for both projects to clash was significant. However, China has thus far demonstrated a degree of restraint, recognizing Russia as an equal and mostly limiting its security-related engagement in the region to ‘addressing direct threats to Xinjiang province, rather than increasing its security presence in Central Asia more broadly’ (Stronski and Ng 2018). Russia, for its part, ‘hopes that its acceptance—though late and grudging—of China as the inevitable future economic hegemon of Central Asia will make it easier for Moscow to stay in the region as a political power and security provider’ due to the Central Asian states’ desire not to be overly dependent on Beijing (Duchâtel et al. 2016).

The ‘porous’ nature of the EAEU, having failed to ‘complete the implementation’ of its rules and norms, has allowed China’s economic presence in Central Asia to proceed largely unimpeded, thus tempering the likelihood of both powers’ key regional interests clashing (Kaczmarski 2019b). This suggests that the complementarity of the two projects has occurred almost accidentally. This situation is unlikely to be helped by the expansion of the SCO to include India and Pakistan as full members, diluting the club and thus making it more implausible that it could serve as a platform where the EAEU and BRI could be harmonized. Bolt and Cross (2018: 96) note that the economic dimension of the Sino-Russian relationship has yet to reach the strategic level, although this could change if Western sanctions against Russia endure for a prolonged period. Nonetheless, competition between the BRI and the EAEU is limited by the differing nature of the two projects: the former is centred on ‘logistics’ and transport, whereas the latter is a customs and regulatory union (Bolt and Cross 2018: 172). Or as Kaczmarski (2019b) puts it, ‘[t]he Chinese elite understands regionalism in functional terms, while its Russian counterpart frames regional cooperation spatially’.

## Russia, China and international society

The above analysis suggests that while certain elements of the Sino-Russian partnership remain instrumental or underdeveloped, there nonetheless remains a relationship between them rooted in nominally shared norms. The return of great power rivalry at the global level has strengthened the rapprochement between the two powers, while the potential for clashing interests and identities at the regional level is less extensive than often assumed. This suggests that fundamental contestation of a normative nature between Moscow and Beijing is unlikely to occur as an impediment to the construction of a thick relationship between them, even if the former occasionally emphasizes the multi-vectored and independent nature of its foreign policy (Petersson 2013: 11; Lukin 2018: 73). As Trenin (2020) notes, a desire for equilibrium in Russian foreign policy is not equivalent to the maintaining of equidistance between China and other leading international actors. This largely ‘optimistic’ forecast is further buttressed by several factors of a more theoretical and conceptual nature.

First is the importance of norms in the English School tradition. While it is possible that both Russia and China have been brought together in large part due to their shared opposition to the excesses of American and Western hegemony, the normative facets of their bilateral relationship could take on a life of their own. A shared set of norms and understandings rooted in a broadly similar conception of global and regional order could become entrenched. For example, Sakwa (2017: 42) notes that following the end of the Cold War, the Western-led liberal international order adopted ‘some sort of tutelary relationship’ with global international society, suggesting that the order had evolved to possess a degree of agency—or at least to operate semi-independently from its constituent states.^6^ It stands to reason that such a process could occur in Sino-Russian relations as well, particularly if the Russia–West relationship remains largely hostile.

Second, although Russia’s ‘pivot to the east’ is in large part associated with a desire to balance against Western hegemony, this does not necessarily imply that sustained and deeper interaction with Eurasia and the Asia–Pacific have not become increasingly meaningful to Russia in their own right. Moreover, if the Sino-Russian relationship remains rooted in the principle of mutual respect and non-interference, then Russia’s insistence on being accorded great power status will not necessarily inhibit their ability to cooperate effectively. To a certain extent, then, the question is not whether Russia remains preoccupied with being accorded great power status, but rather how it chooses to interpret that status. Instead of pushing for an entrenched sphere of influence and promoting a Yalta-type order (Popescu 2020), it could simply be that Russia seeks recognition of its equal status to other great powers, in part through an acknowledgement that its interests are legitimate. According to this reading, it was normative incongruities and the zero-sum nature of Russia–West relations in their shared neighbourhood in Eastern Europe that are largely to blame for the current standoff.^7^ Furthermore, the notion advanced by recent English School scholars that contestation is a driver of international society rather than an inhibitor of its smooth functioning underscores the intersubjective and co-constitutive nature of interstate interaction. Russia and China have been forced to co-opt some of the solidarist language pushed by Western countries in the post-Cold War era to garner international support for their positions, in line with Sakwa’s contention (2017: 43) that the desire of Moscow and Beijing is to ‘universalize universalism’.^8^ This joint embrace of elements of solidarism, as illustrated above, renders it easier for the two powers to construct a thick relationship.

Finally, it is possible at first glance to contend that Russia and China possess different conceptions of international order. Russia, having been relegated to second-tier status in the post-Cold War order, privileges a polycentric conception of order and takes direct aim at the notion of Western leadership, whereas China is more preoccupied with preserving the international conditions conducive to it continuing its economic development and modernization since it has largely benefitted from the international status quo (Kofman 2018; Kaczmarski 2019a; Stronski and Ng 2018). However, upon closer inspection, the core visions of both countries are less incompatible than it might seem. Despite the aforementioned close relationship between international order and international society identified by recent English School scholarship, these two concepts are not entirely synonymous. Flockhart (2016) notes that global international society has not always overlapped with a single international order—such as during the Cold War—and contends that we are now witnessing the emergence of a ‘multi-order’ world. And as mentioned above, Sakwa (2017: 44) asserts that the global political system is composed of two distinguishable realms: a global international society featuring a series of primary and secondary institutions, in addition to a series of competing international orders of bounded geographical scope.

Russia has been a participant European international society for several centuries.^9^ Although its situation at Europe’s periphery can produce elements of dissatisfaction and contestation with the core (Neumann 1995: 206), this has nonetheless allowed Moscow to be a strong rhetorical defender of international society’s primary institutions such as the balance of power and international law, as well as secondary institutions including the United Nations (Sakwa 2017: 47). China’s interaction with European international society took place much later and occurred under very different circumstances, beginning in the nineteenth century but only truly opening up under Deng Xiaoping (Shambaugh 2013: 309).^10^ Since the post-Mao era, it has been undergoing a process of ‘de-alienation’ from an international order and society that was ‘grudgingly regarded as acceptable, though not desirable and fully justifiable’ (Zhang 1999: 126). Whether great power status is sought by Russia for identity-related reasons or merely instrumental purposes—whether to provide its population with a sense that they have a ‘special mission’ or to secure a seat at the table of great powers before its decline becomes too advanced (Richter 1996: 81)—the goal is to bring about a polycentric system for managing global affairs rather than a bipolar one (Kofman 2018). This aim deals with the configuration of the international *order* rather than international society. Moscow’s preoccupation with defending the autonomy of global international society from perceived Western transgressions (Sakwa 2017: 47) can therefore be thought of as an expression of proceduralism and ‘constitutionalism’—an interest in determining who makes the rules rather than what the rules themselves are (Allison 2013: 203; Sokov 2018; Sushentsov and Wohlforth 2020). By contrast, China’s preference for the norm of non-interference and its calls for the ‘democratization’ of international relations are an indication of a conservative and cautious approach to pursuing its ‘de-alienation’ from international *society*. In other words, the precise configuration that the international order takes matters less to Beijing than the need simply to preserve the international conditions necessary for it to complete its national economic and development-related goals (Deng 2005: 66). Far from differing conceptions of international order potentially generating irritants in the Sino-Russian bilateral relationship, Russia’s preoccupation with questions of international order and China’s co-constitutive process with international society represent phenomena of a different nature. Even if the glue of shared opposition to the excesses of American hegemony ceases to bind Russia and China together as Washington gradually redefines its global role, this fact bodes well for the long-term sustainability of their increasingly deep ties. Any future improvement in Russia–West relations may alter the character of global order, moving it away from renascent great power rivalry, but this will not necessarily undo recent social developments in the Moscow–Beijing relationship.

The nature of the Sino-Russian partnership is such that they ‘stay away from contesting each other’s core interests or supporting adversaries in key contests’ (Kofman 2019), even if ‘China has made it very clear that it is not prepared to offer Russia uncritical political support, and is wary of being dragged into other countries’ confrontations where it has no clear stake’ (Duchâtel et al. 2016). While this mixed picture could cause some to conclude that the relationship between Moscow and Beijing is merely instrumental and weak, this could in fact be representative of what some call a ‘new type of great power relationship’ (Bolt and Cross 2018: 15), emphasizing mutual respect and cooperation while compartmentalizing disagreements. Given their currently shared interest in balancing against the West, defence cooperation between Russia and China may grow to the point where their relationship in their shared neighbourhood comes close to being de-securitized (Kofman 2019). This is noteworthy, as Buzan (2012: 39) characterizes de-securitized communities as lying between the ‘Cooperative’ and ‘Convergent’ realms—in other words, as veering strongly towards the solidarist side of the spectrum, beyond mere ‘thick’ pluralism. At the very least, the Russia–China rapprochement in the military sphere poses a greater strategic dilemma for Washington than it does for Moscow (Kashin 2019). And while Moscow remains weary of being relegated to second-tier status behind Washington and Beijing in the global power hierarchy (Kaczmarski 2015: 125–126), China remains more predisposed to cultivating relations carefully with both the USA and Russia rather than forging a ‘G2’ with the USA (Bolt and Cross 2018: 301).

## Conclusion

The level of shared norms and identity between Moscow and Beijing—beyond anti-hegemonism and authoritarianism—may remain somewhat limited. Moreover, it is possible that identity-related developments in both countries, including but not limited to rising nationalism, could drive them apart over the medium-to-long term despite their current normative rapprochement (Rozman 2014: 267–277). But so long as China continues to demonstrate a modicum of restraint in its relations with Russia to compensate for its rising power, the foundations on which their relationship rests are likely to remain stable (Paikin et al. 2019).^11^ As leading global powers, the thick nature of their relationship is likely to shape the contours of global order (Bolt and Cross 2018: 301) and—by extension—the fledgling Eurasian order as well, whether limited to Central Eurasia and the Indo-Pacific or encompassing the entirety of the supercontinent. However, whether their partnership will act as a magnet drawing other actors into a society replete with shared norms or as a repellant encouraging further zero-sum security competition remains to be determined.

The possibility of viewing the Sino-Russian relationship as ‘thick’ from an English School perspective contrasts with more established views. The conception of thin and thick orders advanced by Mearsheimer (2019) would posit that Russia–China ties are thin, in part due to the SCO’s continued inability to complement its security-oriented focus with a substantial economic dimension (Dovgalyuk 2019). A history of mutual suspicion and Russia’s centuries-old European orientation leave sceptics predisposed to doubting the durability of the Sino-Russian strategic partnership (Lo 2008: 194–196; Kortunov 2018). This article has pushed back against such assertions, demonstrating how Moscow and Beijing have been able to forge a thick relationship despite possessing differing aims and an inchoate bilateral normative dialogue. Such a conclusion should challenge Western scholars and policymakers not only to rethink the contours and impact of the Russia–China relationship, but also to dwell on the possibility for cooperative relations to emerge between great powers and regional orders in a global international society marked by normative divergence.^12^

## Footnotes

1 The term ‘political system’ is used here as a term encompassing both international society and international order. As such, the global political system consists of both global international society and the global order that prevails at a given time.

2 The English School traditionally assumes that three competing modes of interaction interplay with one another within the global political system, with the equilibrium between them varying across time and space: international system (Machiavellian/Hobbesian logic), international society (Grotian logic) and world society or community (Kantian logic). See Linklater and Suganami (2006: 117–118).

3 The consolidation of the sovereign state as one would recognize it today following the Napoleonic Wars rather than in 1648 is a notion that has been significantly developed, as is the added speed and scope of the expansion of European international society across the globe following the Industrial Revolution. See Teschke (2003) and Watson (1984).

4 Buzan (2014: 163–165) further notes that solidarism represents an empirical story and not just a normative one. Economic and environmental cooperation, among other examples, represent instances in which a universal solidarism has taken shape at the global level. This, in addition to the spread of solidarist norms and institutions from the West to the ‘rest’ in recent centuries described above, ensures the presence of at least some solidarist content in international social relationships lying outside the liberal West.

5 Definitions of international society advanced by both early and recent English School scholars rely on both norms and identities. See Buzan (2012: 36) and Wight (1977: 13).

6 The semi-independence of norms is also highlighted by Worth (2015: 107), who contends that the current global neoliberal order is not the direct result of a contrived American strategy but rather the by-product of American hegemony.

7 For more on how relations between Russia and the USA gradually adopted a zero-sum character on the question of Europe’s security order, see Stent (2014).

8 This can take the shape, for example, of their joint defence of international law and institutions, in addition to their repurposing of the term democracy to suit their own ends (e.g. ‘sovereign democracy’ or ‘democratization of international relations’). Morozov (2015: 23) highlights this process, albeit to explain Russia’s subaltern normative and ideational dependence on the West.

9 Even recent English School accounts that de-emphasize global international society’s European origins do not list Russia as one of international society’s cultural origin points. Phillips (2017: 43) lists these as Latin Christendom, the Sinosphere and the Islamicate, suggesting that Russia’s inclusion in European international society is a belief shared by competing historical English School accounts.

10 There are two major accounts of how a global international society came about. The first (see Watson 1984) involves a European international society that expanded to encompass the entire globe. The second (see Reus-Smit and Dunne 2017) contends that international society globalized through a polycentric pattern of inter-civilizational interaction. The aim here is not to endorse either narrative—‘expansion’ or ‘globalization’—but rather simply to acknowledge that China’s first significant interaction with European international society occurred in the nineteenth century.

11 The notion that a hegemon must demonstrate restraint for an international order to remain stable is an idea that enjoys currency not just in the English School. For a liberal account, see Ikenberry (1998–1999) and for a neo-realist one, see Monteiro (2014: 231–232).

12 Korosteleva and Flockhart (2020) begin to address this issue in their work on resilience.
